# Editorial: All 3 Types of Glial Cells Are Important for Memory Formation

**DOI:** 10.3389/fnint.2016.00031

**Published:** 2016-09-27

**Authors:** Leif Hertz, Ye Chen

**Affiliations:** ^1^Laboratory of Metabolic Brain Diseases, Institute of Metabolic Disease Research and Drug Development, China Medical UniversityShenyang, China; ^2^Henry M. Jackson FoundationBethesda, MD, USA

**Keywords:** Aquaporin4 and memory, Astrocytes in memory, Brain rhythms and memory, Fetal alcohol syndrome, Glutamate, glycogen and glucocorticoids in memory, Microglia in memory, Neuro- and glio-transmitters in memory, Traumatic brain injury and memory

This editorial review of the research topic describes effects of the glial cells astrocytes, microglia and oligodendrocytes on memory. This includes their interactions with themselves and with neurons, and changes in their histology, physiology, and metabolism observed during learning.

## Astrocytes

As described in Scheller and Kirchhoff astrocytes express receptors for many transmitters, including glutamate and noradrenaline, as well as for cannabinoids (CB1 and CB2 receptors). Receptor stimulation increases free cytosolic calcium [Ca^2+^]_i_, which is an extremely important signal involved in the activation of gliotransmitter release (see also Zorec et al.; Orellana et al.). CB1 receptors are expressed on virtually all brain cells and are partly responsible for cannabis' addictive properties. The role of this receptor in learning is not known. However, cannabis users display specific behavioral changes, including memory impairment, which is abolished in conditional mutant mice lacking CB1 in astrocytes, but conserved in mice lacking this receptor in glutamatergic or GABAergic neurons (Han et al., 2012). CB1-induced increase of [Ca^2+^]_i_ acts differently when it spreads in a single stimulated astrocyte to distant synapses and when it spreads through gap junctions to distant astrocytes. In the former case, it acts on presynaptic metabotropic glutamate receptors to generate persistent changes in synaptic transmission, and in the latter, the increased [Ca^2+^]_i_ releases glutamate which acts on postsynaptic NMDA receptors. On microglia and endothelial cells, stimulation of CB2 receptors has anti-inflammatory effects (Scheller and Kirchhoff).

Gap junctions are formed by paired connexin hemichannels. As described in Orellana et al. connexin hemichannels and pannexins transport gliotransmitters out of astrocytes. These channels also carry lactate, redox molecules and ions, and in the lateral amygdala blockade of Cx43 hemichannels inhibits consolidation of fear memory (Orellana et al.).

Like neurons, astrocytes also release transmitters from vesicles (Zorec et al.), and this release mode is energetically more effective than release via hemichannels (Guček et al., 2012). As described in Zorec et al. astrocytic gliotransmitter responses modify synaptic plasticity and function in different aspects of learning. Vesicular release from astrocytes in response to transmitter stimulation is slower than neuronal transmitter release, consistent with time-dependent memory formation.

## Microglia

As described in Hristovska and Pascual microglia plays important roles in physiological brain activity although they were previously supposed to operate only under pathological conditions. Neuronal activity, sensory experience and neurotransmission modulate their high mobility, process extension and retraction and secretion of ATP among others. Their great plasticity enables them to sense activity, and to regulate synaptic inputs by the secretion of signaling molecules and pruning of synapses.

## Oligodendrocytes

Yamazaki et al. (2014) demonstrated that oligodendrocytes in white matter respond *in vivo* to neuronal activity with prolonged depolarization of membrane potential, which accelerates axonal conduction and increases the number of compound action potentials and thus facilitates learning.

## Glial effects on brain rhythms via interaction with synapses

Tewari et al. describe in rodents that a single astrocyte can interact with 100,000 synapses and in primates and humans with up to 2 million synapses. This large number makes it impossible to experimentally study the full range of interactions. This problem, however, can be partially overcome by mathematical modeling showing that astrocytes greatly increase the amplitude and frequency of neural oscillations and moreover, that different frequencies are associated with specific aspects of learning (Tewari et al.).

Tewari and Parpura noted that neuronal spiking activity, measured as local field potentials (LFPs), represents averages of synaptic currents. These authors suggested that delta oscillations are astrocyte-dependent, but others (Lee et al., 2014) reported that astrocytes contribute to gamma oscillations and recognition memory. Probable reasons for this discrepancy and the possibility of different rhythms in different cognitive tasks or regions are discussed in Tewari and Parpura.

## Water transport in the brain

As discussed in Szu and Binder aquaporin4 (AQP4) is expressed in astrocytes and controls bidirectional water transport in brain. Although AQP4^−∕−^ mice exhibit only subtle effects on basal synaptic transmission these authors show that AQP4 influences synaptic plasticity, hippocampal long-term potentiation (LTP), long-term depression (LTD), learning and memory. The mechanisms involved are not well understood, but they suggest that in AQP4^−∕−^ astrocytes deficient release of brain-derived neurotrophic factor (BDNF) might impair LTP and LTD. Alternatively, glutamate transporter downregulation, excessive activation of NMDA receptors, reduced activity of the co-localized Kir4.1 channel or decreased astrocytic Ca^2+^ entry are suggested as possible inhibitors of LTP in these animals.

## Metabolism: glycogen, glutamate, lactate, and glucocorticoids

### Glycogenolysis, glutamate, and lactate

In astrocytes glycogenolysis is required for release of ATP as a gliotransmitter (Xu et al., 2014) and for K^+^ uptake (Xu et al., 2013). Moreover, it provides metabolic energy for Ca^2+^ homeostasis (Mūller et al., 2014). All of these effects, including K^+^ uptake (Hertz and Chen, 2016), are important for learning. However, the most detailed account of the roles of astrocytic glycogenolysis in learning is given in the paper by Gibbs: During one-trial aversive training in day-old chickens there are 3 brief periods of transmitter-induced glycogenolysis during the first hour, all of which are prevented by the glycogenolysis inhibitor 1,4-dideoxy-1,4-imino-D-arabinitol (DAB). The first of these periods is triggered by serotonin and necessary for an increase in glutamate content, which precedes release of transmitter glutamate transferred to neurons in the glutamate-glutamine cycle. The next is activated by noradrenaline, with β_2_-adrenergic stimulation activating glycogenolysis, and α_2_-adrenoceptors stimulating simultaneous re-synthesis of glycogen. It probably also activates glutamate synthesis (Hertz et al., 2003), and as discussed by Robinson et al. memory formation is abolished when glutamate transfer from astrocytes to neurons is inhibited. The trigger and purpose of the third glycogenolytic period are unknown, but one function might be to release glutamate-derived lactate to the extracellular space, which is essential for learning (see papers by Steinman et al. and DiNuzzo).

As described in Steinman et al. glycogenolysis is required in rats for hippocampal memory consolidation, LTP and expression of cofilin and other memory-related genes. During glycogenolytic inhibition all of these events can be rescued by lactate administration. Lactate exits astrocytes via monocarboxylate transporters (MCT) 1 and 4 and enters neurons via MCT 2. Blockade of MCT1 or MCT4 expression inhibits memory and associated neuronal gene expressions, showing essential effects of extracellular lactate signaling and/or neuronal lactate uptake. MCT1 and 4 are upregulated by learning, but MCT2 is not (Suzuki et al., 2011; Tadi et al., 2015). Nevertheless, memory in rats is inhibited after MCT2 blockade, implying importance of neuronal lactate uptake, although memory-induced rise in extracellular lactate is modest (Steinman et al.). This suggests that only selected structures are accumulating glycogen-derived lactate, which is consistent with the lack of observed learning-induced down-regulation of MCT2. These structures might be the minute glutamatergic dendritic spines expressing AMPA glutamate receptors. They are enriched in MCT2 co-localized with GluA2/3 (Bergersen et al., 2005), and the actin depolymerizing protein cofilin is required for spine enlargement during early LTP (Rust, 2015). The spines rarely have mitochondria (Bergersen et al., 2005), but MCT2-mediated lactate accumulation and subsequent oxidation to pyruvate would rapidly produce ATP needed for remodeling of actin without interfering with glucose utilization by competition for NAD^+^ (Hertz, 2004). LTP dependence on glycogenolysis has been confirmed in brain slices from young rats (Drulis-Fajdasz et al., 2015). Signaling functions of extracellular lactate are further discussed by DiNuzzo. Stimulation of G_i_-protein coupled hydroxycarboxylic acid (HCA) receptors by D- or L-lactate reduces neuronal activity. In contrast, the noradrenergic neurons of locus coeruleus (LC) are stimulated with EC_50_ ~0.5 mM by astrocyte-released L-lactate, but not D-lactate, in a glycogenolysis-dependent manner, a paradigm-shifting observation.

### Glucocorticoids

Learning may be enhanced by the release of glucocorticoids from the adrenal cortex evoked by moderate acute stress, while chronic or extreme stress impairs cognitive processes as discussed in Pearson-Leary et al. This increase is caused by noradrenaline-mediated release of corticotropin-releasing hormone (CRH), promoting secretion of adrenocorticotropic hormone (ACTH) from the anterior pituitary. Both noradrenaline and glucocorticoids act on neurons, astrocytes, oligodendrocytes and microglia, where they cause release of inflammatory cytokines and immunomodulatory molecules affecting learning.

## Learning impairment

### Exposure to toxic substances

Lead is known to cause learning difficulties, which is caused by glutamine synthetase inhibition by oxidative stress and reduced effectiveness of the glutathione system as described in Robinson et al.

### Fetal alcohol syndrome

As discussed in Wilhelm and Guizzetti alcohol is especially damaging for memory during prenatal exposure. Fetal alcohol spectrum disorders (FASD) lead to a broad spectrum of cognitive and behavioral impairments. Abnormal neuronal and glial migration reflects radial glia impairment, astrocyte proliferation or survival is reduced, and oligodendrocyte development delayed. Disrupted microglial function and survival cause deficient synaptic pruning and abnormal brain plasticity. These glial effects disrupt neuronal development and survival and brain architecture and connectivity.

### Traumatic brain injury (TBI)

Brain trauma causes microglia-induced astrocyte reactivity and secretion of neuroinflammatory molecules that either impairs axonal regrowth and long-term cognitive outcomes or improves neuronal survival and cognitive abilities over time as discussed in Sajja et al. Axonal disruption and oligodendrocyte dysfunction impair neurotransmission by degeneration of white matter tracts. Astrocytic K^+^ channel impairment contributes to cognitive impairment.

### Psychiatric disease

Chen et al. described that whereas astrocytic stimulation of the serotonergic 5-HT_2B_ receptor is essential for learning (Gibbs), down-regulation of the same receptor in these cells by fluoxetine has antidepressant effects, confirming previous findings of a relationship between psychiatric disease and learning.

## Concluding remarks

Glial effects on learning are exerted by many types of physiological and metabolic effects that influence neuronal activities. The two astrocyte-specific processes glutamate synthesis and glycogenolysis are indispensable for all forms of learning.

## Author contributions

All authors listed, have made substantial, direct and intellectual contribution to the work, and approved it for publication.

### Conflict of interest statement

The authors declare that the research was conducted in the absence of any commercial or financial relationships that could be construed as a potential conflict of interest.
